# Production of Hybrid-IgG/IgA Plantibodies with Neutralizing Activity against Shiga Toxin 1

**DOI:** 10.1371/journal.pone.0080712

**Published:** 2013-11-28

**Authors:** Katsuhiro Nakanishi, Sanshiro Narimatsu, Shiori Ichikawa, Yuki Tobisawa, Kohta Kurohane, Yasuo Niwa, Hirokazu Kobayashi, Yasuyuki Imai

**Affiliations:** 1 Laboratory of Microbiology and Immunology, School of Pharmaceutical Sciences, University of Shizuoka, Shizuoka City, Shizuoka, Japan; 2 Laboratory of Plant Molecular Improvement, Graduate Division of Nutritional and Environmental Sciences, University of Shizuoka, Shizuoka City, Shizuoka, Japan; 3 Graduate Program in Pharmaceutical and Nutritional Sciences, University of Shizuoka, Shizuoka City, Shizuoka, Japan; Wadsworth Center, New York State Dept. Health, United States of America

## Abstract

Shiga toxin 1 (Stx1) is a virulence factor of enterohemorrhagic *Escherichia coli*, such as the O157:H7 strain. In the intestines, secretory IgA (SIgA) is a major component of the immune defense against pathogens and toxins. To form SIgA, the production of dimeric IgA that retains biological activity is an important step. We previously established hybrid-IgG/IgA having variable regions of the IgG specific for the binding subunit of Stx1 (Stx1B) and the heavy chain constant region of IgA. If hybrid-IgG/IgA cDNAs can be expressed in plants, therapeutic or preventive effects may be expected in people eating those plants containing a “plantibody”. Here, we established transgenic *Arabidopsis thaliana* expressing dimeric hybrid-IgG/IgA. The *heavy* and *light chain* genes were placed under the control of a bidirectional promoter and terminator of the chlorophyll *a/b*-binding protein of *Arabidopsis thaliana* (expression cassette). This expression cassette and the *J chain* gene were subcloned into a single binary vector, which was then introduced into *A. thaliana* by means of the *Agrobacterium* method. Expression and assembly of the dimeric hybrid-IgG/IgA in plants were revealed by ELISA and immunoblotting. The hybrid-IgG/IgA bound to Stx1B and inhibited Stx1B binding to Gb_3_, as demonstrated by ELISA. When Stx1 holotoxin was pre-treated with the resulting plantibody, the cytotoxicity of Stx1 was inhibited. The toxin neutralization was also demonstrated by means of several assays including Stx1-induced phosphatidylserine translocation on the plasma membrane, caspase-3 activation and 180 base-pair DNA ladder formation due to inter-nucleosomal cleavage. These results indicate that edible plants containing hybrid-IgG/IgA against Stx1B have the potential to be used for immunotherapy against Stx1-caused food poisoning.

## Introduction

Enterohaemorrhagic *Escherichia coli* (EHEC) strains such as O157:H7 are important human food-borne pathogens [1], [2]. EHEC and *Shigella dysenteriae* infect more than 150 million people each year and cause more than a million deaths [3]. EHEC causes a variety of symptoms from watery diarrhea to hemorrhagic colitis, and severe complications such as haemolytic-uraemic syndrome (HUS) that can cause death in up to 5% of HUS cases [4], [5]. There is no effective treatment because large amounts of Shiga toxins (Stx), virulence factors produced by EHEC, are released from EHEC on treatment with antibiotics [4], [6]. Two types of Stx, Stx1 and Stx2, are known to be associated with human diseases [7], [8]. Of them, Stx1 is also produced by *Shigella dysenteriae.* Stx consists of a cytotoxic A subunit and a pentamer of cell-binding B subunits [9]. The A subunit inhibits protein synthesis due to its *N*-glycosidase activity, which removes an adenine base from 28S ribosomal RNA and induces apoptotic cell death [9], [10]. The B subunits bind to cell surface carbohydrate ligands like globotriaosylceramide (Gb_3_), which is also known as CD77 in human activated B-lymphocytes [11], [12]. CD77 is expressed on some Burkitt’s lymphoma cell lines and germinal center B cells in humans, but not on mouse germinal center B cells [13]–[15]. Gb_3_ is also expressed on Vero cells, which are derived from the kidneys of African green monkeys, and Stx exhibits cytotoxicity toward Vero cells [16].

Immunoglobulin A (IgA) is one of the major factors for immune defense against pathogens and toxins on mucosal surfaces such as that of the intestines [17]. On the mucosal surface, IgA is secreted as SIgA consisting of dimeric IgA (dIgA), comprising two IgA monomers covalently linked through a joining (J) chain, and a secretory component (SC). By binding with SC, IgA gains resistance to digestive enzymes and the ability to be localized near the epithelial surface through anchoring to the mucus [18]–[20]. Thus, SIgA is supposed to function well in the protection of the gastrointestinal tract. Based on this function, SIgA is expected to prevent infectious diseases by excluding the entry of toxic substances and pathogens from the gastrointestinal tract when used as an orally administered therapeutic antibody.

Production of therapeutic antibodies using mammalian cell cultures has limitations due to the high cost and limited scalability of production [21], [22]. A plant expression system is expected to be a candidate for solving these problems because of the lower cost of production and higher scalability [21]–[25]. Plants are also suitable as hosts for the production of edible pharmaceutical proteins [26]–[28]. EHEC can be transmitted by fruits and vegetables contaminated with the faeces of domestic or wild animals [29]. Thus, physical containment is required, but this is naturally a part of the production process for transgenic plants. Because it does not require sterile syringes and health professionals, the oral administration of therapeutic proteins will be particularly useful in developing countries. If Stx-specific SIgA can be expressed in plants, therapeutic or preventive effects would be expected in people eating these plants expressing recombinant antibodies. Recombinant antibodies produced by plants have been proposed to be called “plantibodies” [23].

In previous studies, we established mouse hybridoma cell lines producing IgA and IgG monoclonal antibodies (mAb) against Stx1B [30], [31]. Of the two, the IgG mAb more efficiently inhibited Stx1B binding to cell surface ligands. To produce IgA with stronger biological activity, we developed hybrid-IgG/IgA cDNAs with variable regions of IgG mAb and the heavy chain constant region of IgA mAb [32]. Upon expression in COS-1 cells, we found that the hybrid-IgG/IgA inhibited Stx1B binding to Gb_3_/CD77-positive Ramos cells.

In this study, we generated transgenic *Arabidopsis thaliana* expressing hybrid-IgG/IgA. In order to produce SIgA in plants, the production of functional dimeric IgA is an important step, because monomeric IgA without the J chain can not form SIgA. Therefore, we focused on the production of biologically active dimeric IgA in plants. The hybrid-IgG/IgA plantibody retained Stx1B binding activity and neutralized Stx1 holotoxin *in vitro*. These results indicate that edible plants containing hybrid-IgG/IgA against Stx1B can be created as a possible means of immunotherapy against Stx1-caused food poisoning.

## Materials and Methods

### Vector construction

As terminators, T*_CAB1_* and T*_CAB2_* (positions 15,424 to 16,075 and 19,906 to 20,768, respectively, derived from the FIN18 BAC clone, accession number AC008030) were isolated from *A. thaliana* genomic DNA by PCR using specific primer sets, i.e., TCAB1 fd *Not* and TCAB1 rv *Nsi* or TCAB2 fd *Sac* and TCAB2 rv *Nsi*, which are listed in Table S1. Amplified DNA fragments were inserted into the pCR®4-TOPO® TA cloning vector (Invitrogen, Carlsbad, CA, USA). Following *Not*I/*Nsi*I or *Sac*I/*Nsi*I digestion, the T*_CAB_* terminators were ligated into the pGEM5zf vector (Promega, Madison, WI, USA). Plasmids having one of the terminators were digested with *Not*I, and then ligated with *Not*I-digested DNA fragments of the Stx1B-specific hybrid-IgG/IgA Hc or Lc cDNA [32], resulting in the production of pGEM5zf/Hc-T*_CAB1_* and pGEM5zf/Lc-T*_CAB2_*. The Hc-T*_CAB1_* and Lc-T*_CAB2_* DNA fragments were excised from pGEM5zf/Hc-T*_CAB1_* and pGEM5zf/Lc-T*_CAB2_*, respectively, with *Apa*I/*Hin*dIII, and then inserted into the *Hin*dIII site of pGEM3zf (Promega) by means of three-piece ligation, resulting in the production of pGEM3zf/T*_CAB1_*-Hc-Lc-T*_CAB2_*. As a promoter, P*_CAB_* (positions 16,915 to 19,092 in the FIN18 BAC clone) was isolated from *A. thaliana* genomic DNA by PCR using a primer set, i.e., PCAB2F2-*Sac*II and PCAB1R2-*Sac*II. The resulting DNA fragments were treated with *Sac*II and inserted into *Sac*II-digested pGEM3zf/T*_CAB1_*-Hc-Lc-T*_CAB2_* to create the T*_CAB1_*-Hc-P*_CAB_*-Lc-T*_CAB2_* DNA fragments termed the hybrid-IgG/IgA expression cassette (pGEM3zf/hybrid-IgG/IgA expression cassette). Mouse J chain cDNA (accession number AB644392) [32] was amplified by PCR using JCF-*Xba* and JCR-*Xho*. The amplified DNA fragments were treated with *Xba*I/*Xho*I and subcloned into the *Xba*I/*Xho*I-digested pBCH1 binary vector [33], resulting in the production of pBCH1/Jc. The hybrid-IgG/IgA expression cassette was excised from the pGEM3zf/hybrid-IgG/IgA expression cassette by digestion with *Hin*dIII, and then inserted into the *Hin*dIII site of pBCH1/Jc, resulting in the production of pBCH1/dimeric hybrid-IgG/IgA.

### Transformation of Arabidopsis thaliana

The pBCH1/dimeric hybrid-IgG/IgA was introduced into *Agrobacterium tumefaciens* GV3101 by electroporation using a Gene Pulser II (Bio-Rad, Hercules, CA, USA). Wild-type *A. thaliana* (ecotype Col-0) plants were grown in a temperature-controlled room with 24 h light at 20°C for 6 wk. *A. thaliana* plants were transformed via the floral dip method as described previously [34]. For efficient transformation, the floral dip procedure was performed 3 times at intervals of 3 or 4 d. The primary transformants were selected on MS medium supplemented with 20 µg/ml hygromycin B. After selection, hygromycin B-resistant *A. thaliana* plants were transferred to soil in pots and then grown under the same conditions as for the wild-type plants. The recombinant plants were grown within the physical containment level 1-plant (P1P) facility of the University of Shizuoka.

### DNA analysis

Genomic DNA was extracted from the leaves of transgenic *A. thaliana* plants by boiling in a DNA extraction buffer (100 mM Tris-HCl [pH 9.5], 1 M KCl, 10 mM EDTA). PCR was performed with KOD FX Neo (TOYOBO, Osaka, Japan) using the following specific primer sets: IgG Heavy *Not*F and IgA-H/*Not*R for the heavy chain; IgGk *Not*F and IgGk *Not*R for the light chain; JCF-*Xba* and JCR for the J chain; and actin2-F and actin2-R for a house keeping gene *ACTIN2* of *A. thaliana*.

### mRNA analysis

Total RNAs were extracted from the leaves of transgenic plants using an RNeasy Mini Kit (QIAGEN, Hilden, Germany). RT-PCR was performed with an AccessQuick RT-PCR System (Promega) using the same gene-specific primer sets as for DNA analysis.

### Protein extraction

Leaves of transgenic *A. thaliana* plants were ground in liquid nitrogen to a fine powder with a mortar and pestle. Soluble proteins were extracted with a protein extraction buffer (10 mM MOPS-KOH [pH 7.5], 150 mM NaCl, 0.5 mM EDTA, protease inhibitor cocktail for plant cell and tissue extracts [Sigma-Aldrich, St. Louis, MO, USA]). Cell debris was removed by centrifugation (21,500 × g, 10 min, 4°C), and the supernatant was used as a source of plantibodies. The protein concentrations in the extracts were determined with a BCA protein assay kit (Thermo Scientific, Rockford, IL, USA) using BSA (Thermo Scientific) as a standard.

### SDS-PAGE and immunoblotting

Total soluble proteins were separated by SDS-PAGE on a 7.5% gel (non-reducing conditions; Mini-PROTEAN® TGX™ Precast Gels #456-1026, Bio-Rad) and a 12% gel (reducing conditions; Mini-PROTEAN® TGX™ Precast Gels #456-1046, Bio-Rad), and then electrically transferred to a PVDF membrane. Hybrid-IgG/IgA were detected with an SNAP i.d. Protein Detection System (Millipore, Billerica, MA, USA) with HRP-goat anti-mouse IgA (α chain-specific) (1∶500 dilution; SouthernBiotech, Birmingham, AL, USA), goat anti-mouse kappa (2 µg/ml; SouthernBiotech) plus HRP-donkey anti-goat IgG (0.4 µg/ml; Santa Cruz Biotechnology, Santa Cruz, CA, USA), or rabbit anti-mouse J chain (1 µg/ml; Santa Cruz Biotechnology) plus HRP-goat anti-rabbit IgG (1∶500 dilution; Zymed, South San Francisco, CA, USA). The signals representing the heavy, light and J chains were enzymatically detected using a chemiluminescence reagent, West Pico (Thermo Scientific). As molecular weight standards, HiMark™ Pre-Stained High Molecular Weight Protein Standards (Invitrogen) and MagicMark™ XP Western Protein Standards (Invitrogen) were used. Expression of monomeric and dimeric hybrid-IgG/IgA in COS-1 cells was carried out as previously described [32]. The methods for the expression of monomeric hybrid-IgG/IgA in *A. thaliana* are described in a Supporting Information file (Method S1). Proteins were extracted as described above.

### ELISA

To determine the total hybrid-IgG/IgA in samples, a sandwich ELISA was performed [32]. The hybrid-IgG/IgA in the samples was captured with goat anti-mouse kappa that had been coated (100 ng/well; SouthernBiotech) onto the wells of an ELISA plate (Costar 9018; Corning, NY, USA), and the captured antibodies were detected with HRP-goat anti-mouse IgA (α chain-specific) (1∶1,000; SouthernBiotech). Mouse IgA myeloma protein TEPC 15 (Sigma-Aldrich) was used as a standard. The samples and antibodies were diluted in PBS containing 0.1% bovine serum albumin (BSA) and 0.1% Tween-20. As a substrate, 100 µl of 1 mM 2,2′-azinobis (3-ethylbenzothiazoline-6-sulphonic acid) dissolved in 0.1 M citrate buffer (pH 4.2) containing 0.03% H_2_O_2_ was added, and absorbance readings were made with a microplate reader (SUNRISE Rainbow RC-R; Tecan, Salzburg, Austria) at 405 nm. Binding of plantibodies to immobilized Stx1B (500 ng/well) was quantitated by incubation with HRP-goat anti-mouse IgA (α chain-specific) (1∶1,000; SouthernBiotech) or goat anti-mouse kappa (1 µg/ml; SouthernBiotech) plus HRP-donkey anti-goat IgG (0.4 µg/ml; Santa Cruz Biotechnology), as described previously [32]. The inhibitory effect on the binding of Stx1B to Gb_3_ was assessed by means of an ELISA as described previously [35]. A solution of Gb_3_ (200 pmol/well; Nacalai, Kyoto, Japan) dissolved in methanol (50 µl) was added to each well of an ELISA plate (MaxiSorp; Nunc, Roskilde, Denmark), and then the wells were allowed to dry to immobilize Gb_3_. Binding of digoxigenin-conjugated Stx1B (25 ng/ml; DIG-Stx1B), which had been pretreated with or without the plantibody for 1 h, to the immobilized Gb_3_ was determined with HRP-sheep anti-digoxigenin Fab fragments (1∶500 dilution; Roche Diagnostics, Basel, Switzerland). Recombinant purified Stx1B and DIG-Stx1B were prepared as described previously [35].

### Immunohistochemistry

*A. thaliana* leaves were fixed in 2% (vol/vol) glutaraldehyde in 0.1 M potassium phosphate buffer (pH 7.2) for 2 h at room temperature. The samples were embedded in OCT compound (Sakura Finetek, Tokyo, Japan) and then frozen in liquid nitrogen. Cryostat sections (10-µm thick) were made. The sections were blocked with 3% BSA, and then incubated with goat anti-mouse IgA (α chain-specific) (2 µg/ml; Zymed) or purified goat IgG (2 µg/ml; Zymed) diluted in PBS containing 0.3% BSA for 30 min. The bound antibodies were detected by incubation with alkaline phosphatase-labeled rabbit anti-goat IgG (2 µg/ml; Zymed) for 30 min. The sections were stained with a BCIP/NBT Alkaline Phosphatase Substrate Kit IV (Vector, Burlingame, CA, USA).

### Cell viability assay

To test toxin neutralization, Stx1-sensitive Vero cells were employed [31]. Vero cells (American Type Culture Collection; Rockville, MD, USA) were incubated with 20 pg/ml of Stx1 holotoxin that had been pre-treated with or without the plantibody for 1 h. After 48 h incubation at 37°C under a humidified atmosphere of 5% CO_2_/95% air, cell viability was measured by means of a colorimetric assay using a Cell Counting Kit-8 (DOJINDO, Kumamoto, Japan). Absorbance readings were made at 450 nm with reference at 650 nm. Viability was defined as the percentages of absorbance readings for the Stx1-treated cells relative to those for untreated cells.

### DNA fragmentation assay

The DNA fragmentation assay was performed as described previously [31]. Vero cells were placed in the wells of a 6-well culture plate (Falcon 3046; BD, Franklin Lakes, NJ, USA) at 1.5×10^6^ cells per well and cultured for 16 h. Stx1 that had been pre-incubated with the plantibody for 1 h at 37°C was added to Vero cells to a final concentration of 10 pg/ml. After 48 h incubation, the cell monolayer was washed with 0.02% EDTA–PBS, and then cells were recovered by treatment with 0.25% Trypsin–0.02% EDTA–PBS. After washing in PBS, the cells were lysed with 0.5% Triton X-100 containing 10 mM Tris–HCl (pH 7.4) and 10 mM EDTA for 10 min on ice. The cell lysates were centrifuged and the supernatants were collected. The supernatants were incubated in 0.2 mg/ml of RNase A (QIAGEN) for 1 h at 37°C, followed by incubation with 0.2 mg/ml of proteinase K (Roche) for 30 min at 50°C, and then the DNA fragments were precipitated with 50% isopropanol containing 0.4 M NaCl at –30°C overnight. The DNA fragments were dissolved in 10 mM Tris–HCl containing 1 mM EDTA, analysed by agarose gel electrophoresis, and then stained with ethidium bromide.

### Activated caspase-3 detection assay

Stx1 holotoxin and appropriately diluted plantibody were mixed and incubated for 1 h at 37°C. The mixture containing 10 pg/ml of Stx1 was added to 2×10^5^ of Ramos cells (American Type Culture Collection; Rockville, MD, USA), followed by incubation for 5 h at 37°C in RPMI 1640 (Nissui Pharmaceuticals, Tokyo, Japan) containing 60 µg/ml kanamycin with 10% fetal bovine serum (Hyclone, South Logan, UT, USA) under a humidified atmosphere of 5% CO_2_/95% air. Activated caspase-3 within the cells was detected with a fluorescent substrate using an APOPCYTO™ Intracellular Caspase-3 Activity Detection Kit (MBL, Nagoya, Japan), and analyzed with an FACSCanto™ II (BD).

### Annexin V binding assay

Stx1 holotoxin and appropriately diluted plantibody were mixed and incubated for 1 h at 37°C. The mixture containing 10 pg/ml of Stx1 was added to 2×10^5^ of Ramos cells, followed by incubation for 5 h at 37°C as shown above. The phosphatidylserine translocation on the plasma membrane was detected with FITC-labeled annexin V using an FITC Annexin V Apoptosis Detection Kit I (BD Pharmingen), and analyzed with an FACSCanto™ II (BD).

## Results

### Generation of dimeric hybrid-IgG/IgA transgenic *A. thaliana*

**Figure 1:**
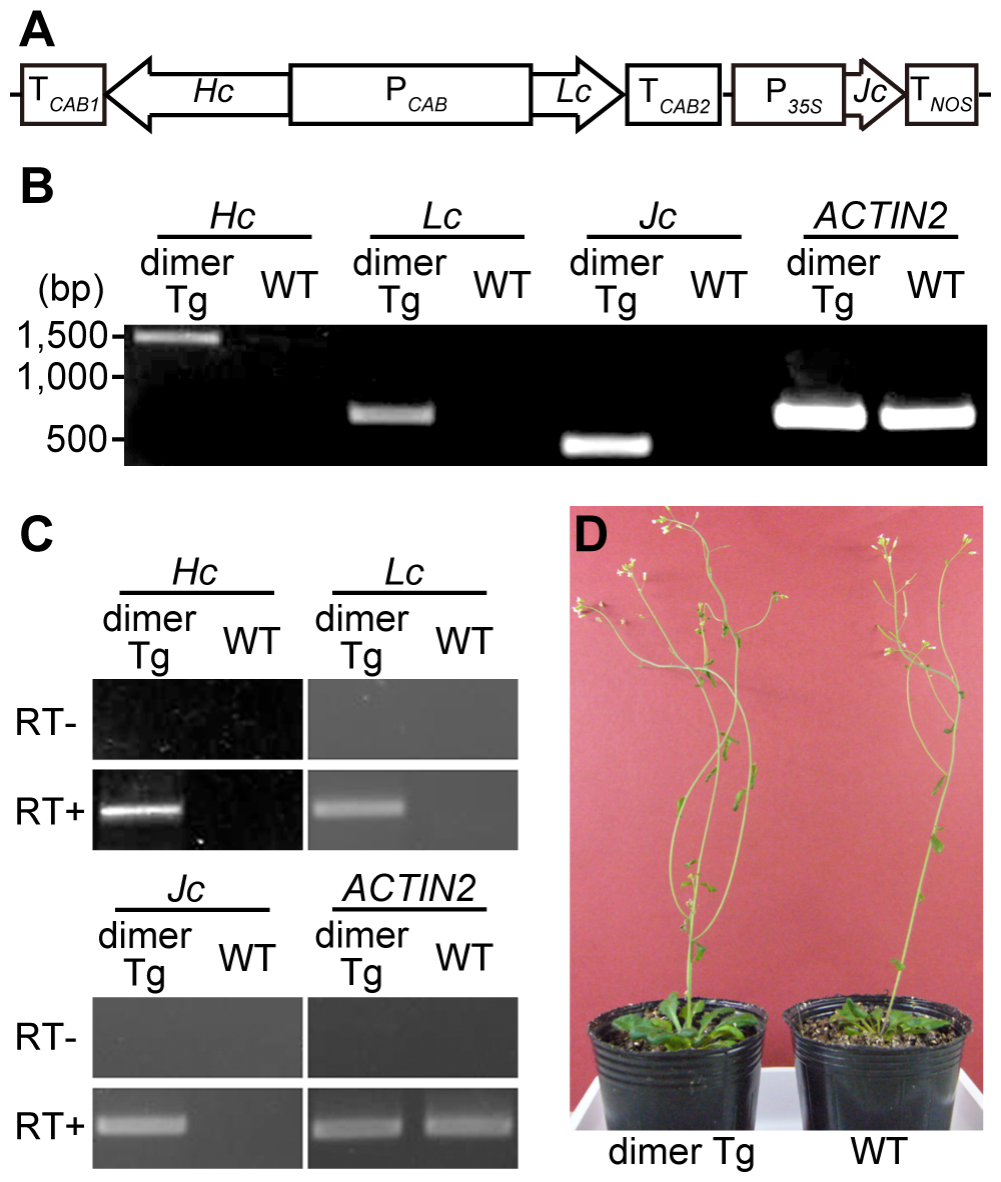
Development of transgenic *A. thaliana*. A. Schematic representation of the dimeric hybrid-IgG/IgA expression vector. P*_CAB_*, chlorophyll *a/b*-binding protein promoter; T*_CAB1_* and T*_CAB2_*, chlorophyll *a/b*-binding protein terminators; *Hc*, hybrid-IgG/IgA heavy chain; *Lc*, hybrid-IgG/IgA light chain; *Jc*, immunoglobulin J chain; P*_35S_*, cauliflower mosaic virus *35S* promoter; T*_NOS_*, nopaline synthase terminator. B. Incorporation of the dimeric hybrid-IgG/IgA transgene into the genome of *A. thaliana*, as revealed by PCR analysis. *ACTIN2* is a house-keeping gene of *A. thaliana*. C. RT-PCR analysis of the mRNA for the dimeric hybrid-IgG/IgA. D. Appearance of a transgenic plant grown on soil for 7 wk. dimer Tg, *A. thaliana* transgenic for H, L and J chains; WT, wild-type *A. thaliana*.

To obtain plants expressing dimeric hybrid-IgG/IgA, an expression vector encoding dimeric hybrid-IgG/IgA was constructed (Figure 1A). A hybrid-IgG/IgA expression cassette was constructed, in which the *hybrid-IgG/IgA heavy* and *light chain* genes were placed under the control of a bidirectional promoter and terminators of the chlorophyll *a/b*-binding protein derived from *A. thaliana* (P*_CAB_*, T*_CAB1_* and T*_CAB2_*). The hybrid-IgG/IgA expression cassette and *J chain* gene were subcloned into the same binary vector, pBCH1, harboring a hygromycin B resistance marker, *HPT* [33]. The *J chain* gene expression was under the control of the *35S* promoter and *NOS* terminator. The resulting dimeric hybrid-IgG/IgA expression vector was introduced into *A. thaliana* through *Agrobacterium*-mediated transformation. On selection on hygromycin B-containing MS plates, five hygromycin-resistant *A. thaliana* plants were obtained. These five transgenic *A. thaliana* lines were transferred to soil and grown to maturity. Genomic PCR analyses revealed that all the transgenic lines had incorporated dimeric *hybrid-IgG/IgA* genes into the plant genome. All the transgenic lines were shown to produce IgA proteins, and the expression levels of IgA in these lines were compared by means of a sandwich ELISA. The line exhibiting the highest IgA expression was selected, and the results of detailed analyses are presented here. DNA fragments corresponding to the heavy, light and J chains were detected in leaves of transgenic but not wild-type plants. A house-keeping gene, *ACTIN2*, was detected in both cases (Figure 1B). RT-PCR analysis was performed to confirm the transcription of the mRNAs for the dimeric *hybrid-IgG/IgA* genes. The heavy, light and J chains were transcribed in the leaves of transgenic but not wild-type plants. The transcription levels of *ACTIN2* were similar in both cases (Figure 1C). The dimer transgenic (dimer Tg) *A. thaliana* was morphologically normal, having the same appearance as wild-type plants (Figure 1D). The morphology of the other four dimer Tg lines was not different from that of the wild-type plants (data not shown).

### Expression and assembly of the dimeric hybrid-IgG/IgA plantibody in transgenic *A. thaliana* leaves

**Figure 2:**
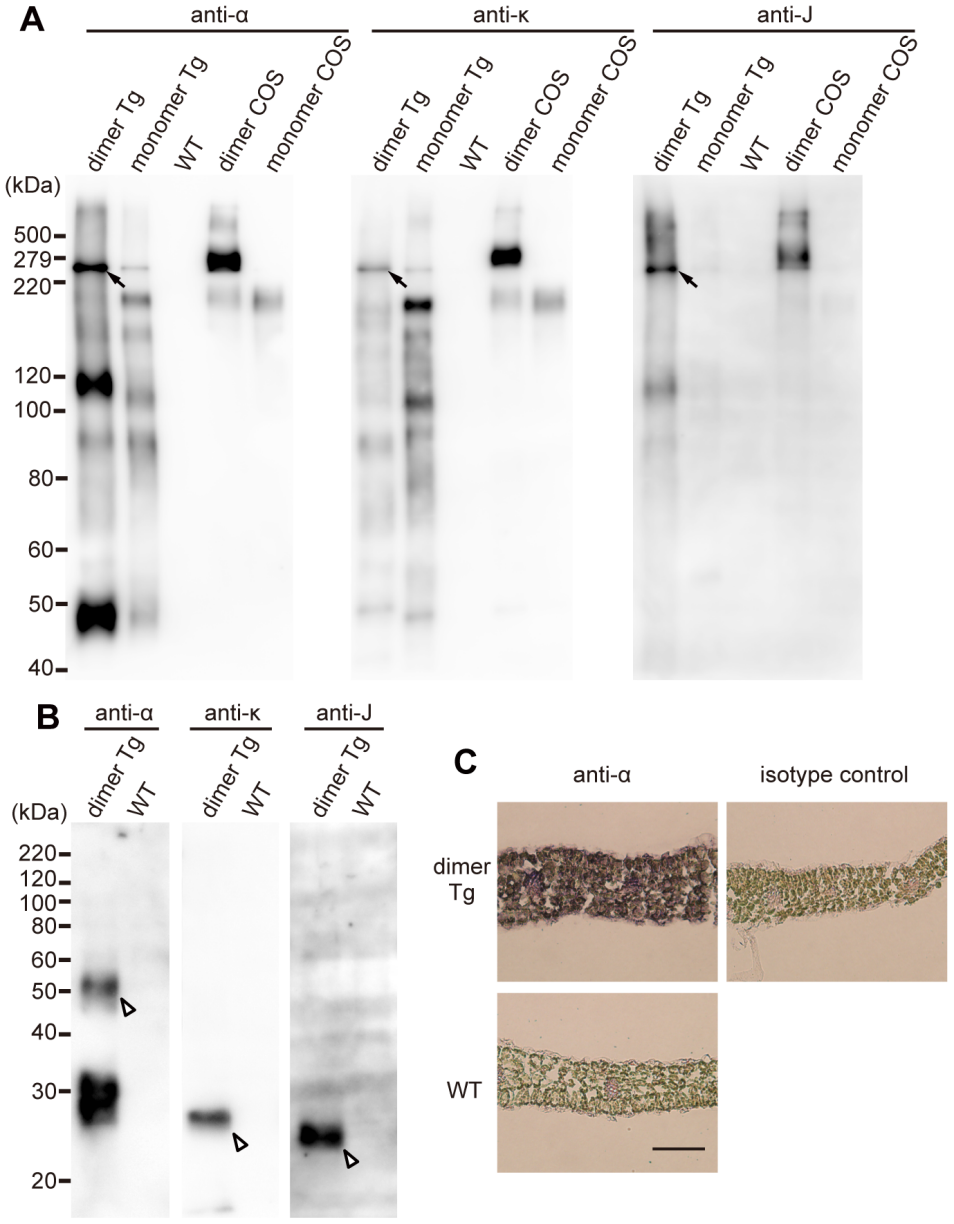
Expression and assembly of the plantibody in *A. thaliana* leaves. A and B. Western blot analysis of proteins extracted from leaves of *A. thaliana*, either transgenic for H, L and J chains (dimer Tg), transgenic for H and L chains (monomer Tg), or wild- type plants (WT). Total protein extracts of leaves were separated by SDS-PAGE under non-reducing (panel A; 7.5% gel; 10 µg protein/lane) or reducing (panel B; 12% gel; 15 µg protein/lane) conditions, and then blotted onto a PVDF membrane. COS-1 cell-derived dimeric (dimer COS) and monomeric (monomer COS) hybrid-IgG/IgA were employed as controls. COS-1-derived hybrid-IgG/IgA proteins were used at 0.5 ng IgA per lane. Each immunoglobulin chain of the hybrid-IgG/IgA was detected with goat anti-mouse IgA (anti-α), goat anti-mouse kappa (anti-κ), and rabbit anti-mouse J chain (anti-J) antibodies. Arrows indicate assembled dimeric hybrid-IgG/IgA (A), and arrowheads indicate each chain after reduction (B). The positions of molecular weight standards are shown on the left in kDa. C. Immunohistochemical analysis of leaf tissue sections. Frozen 10-µm sections of leaf tissue were stained with goat anti-mouse IgA (anti-α) or an irrelevant goat IgG (isotype control). Bars represent 100 µm.

To determine whether or not dimeric hybrid-IgG/IgA was produced, protein extracts of leaves of the dimer Tg plants were subjected to SDS-PAGE, followed by immunoblotting with antibodies specific for the α-, κ- or J-chain. Under non-reducing conditions, a band corresponding to the molecular mass of the dimeric form was detected with all of these antibodies (Figure 2A, arrows) for the protein extracts of transgenic plants expressing the H, L and J chains (dimer Tg). This band exhibits the same electrophoretic mobility as that of dimeric IgG/IgA expressed by COS-1 cells (dimer COS). No such band was detected for wild-type plants, indicating specific expression of the hybrid-IgG/IgA in the transgenic plants. Some other bands of IgA heavy chain epitopes appeared to represent an H chain dimer with a J chain, a heterodimer with H and L chains, and free H chains or their fragments.

For comparison, monomeric IgG/IgA-transgenic *A. thaliana* (monomer Tg) and COS-1 cells transfected with monomeric IgG/IgA constructs (monomer COS) were analyzed. They gave a band corresponding to a smaller molecular mass than that of dimeric IgG/IgA. There was a faint band at the position of dimeric IgG/IgA that may represent spontaneous dimerization in the monomer Tg plants. Consistent with the lack of transformation with J chains, this band was detectable on anti-α and anti-κ immunoblots but not on an anti-J one. Such a band at the position of dimeric IgG/IgA was not observed for COS-1 cells transfected with H and L chains alone. On the other hand, there was essentially no band at the position of a monomer in the case of the transgenic plants expressing H, L and J chains together (dimer Tg).

Under reducing conditions, the α, κ and J chains were detected with antibodies specific for each chain at the expected position for the protein extracts of dimer Tg plants (Figure 2B, arrowheads). No such band was observed under reducing conditions in wild-type plants. An additional band corresponding to around 30 kDa was also observed with anti-α chain antibodies only in the dimer Tg lane, but the identity of this material has not been determined yet. The immunohistochemical staining of leaf sections with anti-α antibodies specifically revealed the protein expression in the dimer Tg but not the wild-type plants (Figure 2C).

**Figure 3:**
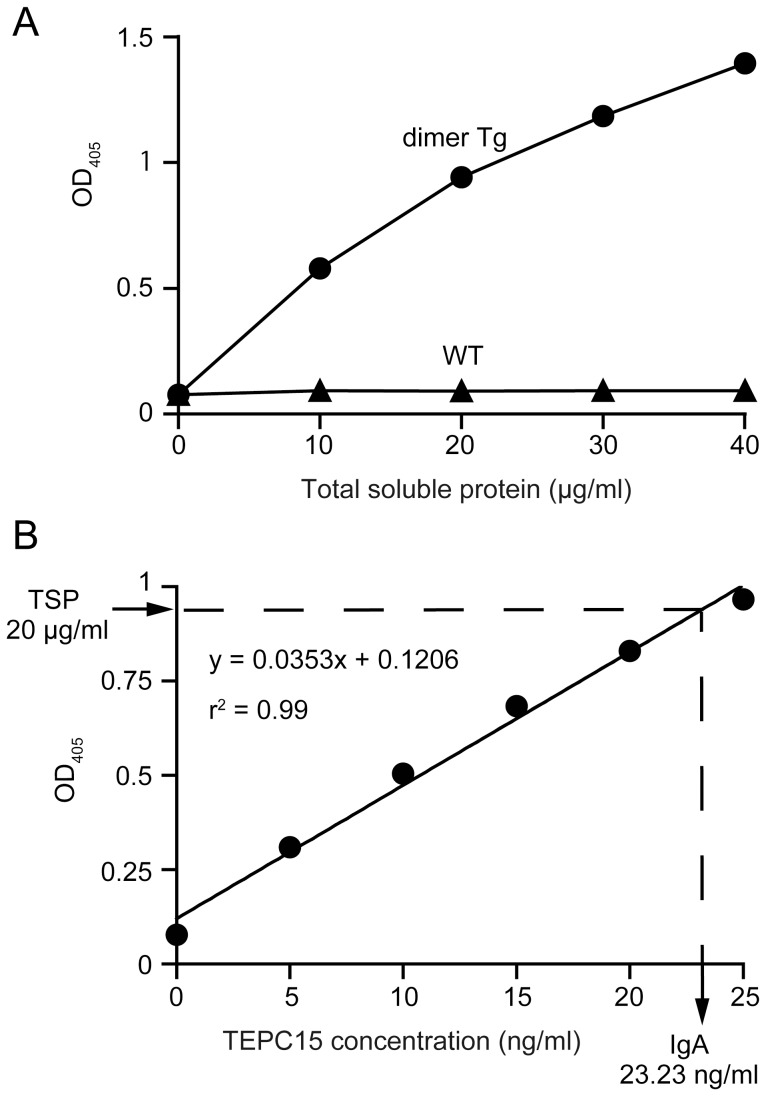
Quantification of assembled hybrid-IgG/IgA on sandwich ELISA. A. Hybrid-IgG/IgA in extracts of transgenic *A. thaliana* leaves, which contained the indicated amounts of total soluble protein (TSP), was captured with immobilized goat anti-mouse κ and then detected with HRP-goat anti-mouse IgA (α chain-specific). The samples were from dimer Tg plants (closed circles) or wild-type plants (closed triangles). B. Standard curve for quantification of IgA. IgA molecules that had been captured by the immobilized goat anti-mouse κ chain were detected with HRP-goat anti-mouse IgA on ELISA. Various concentrations of purified mouse myeloma protein TEPC 15 were used to generate a standard curve. Data are expressed as means ± SD of triplicate determinations. Error bars underneath the symbols are not visible. The results are representative of three experiments.

With the limitations as to impurities, IgA proteins having both H and L chains could be measured by means of a sandwich ELISA, for which the proteins were captured with anti-κ chain antibodies and detected with anti-α chain ones (Figure 3A). Protein extracts of dimer Tg plants gave positive signals in response to total soluble protein (TSP) in leaf extracts whereas those of wild-type plants did not show any signal. We quantified these forms of assembled IgA proteins by ELISA in comparison with purified IgA myeloma TEPC 15 as a standard (Figure 3B). On this quantification, we found that the hybrid-IgG/IgA expression level in dimer Tg plants reached 0.11% of TSP (11 μg/g leaf tissue). Taken together, these results indicate that hybrid-IgG/IgA plantibodies are expressed and assembled into a dimeric form in dimer Tg plant leaves.

### Inhibition of Stx1B binding to Gb_3_ by the plantibody

**Figure 4:**
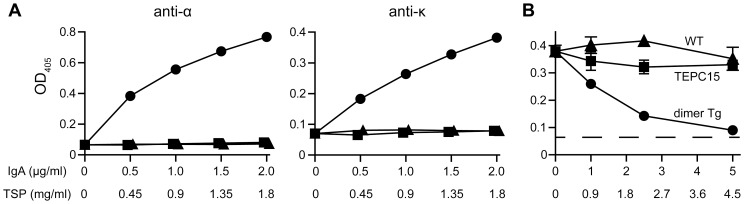
The plantibody binds to Stx1B and inhibits the binding activity of Stx1B. A. Binding of the hybrid-IgG/IgA to immobilized Stx1B. Extracts of dimer Tg plants (closed circles), ones of wild-type plants (closed triangles), or purified mouse IgA myeloma protein TEPC 15 (closed squares) were allowed to bind to immobilized Stx1B. The bound hybrid-IgG/IgA was detected with goat anti-mouse IgA (anti-α) or goat anti-mouse kappa (anti-κ). B. Inhibition of the binding of DIG-Stx1B to immobilized Gb_3_. DIG-Stx1B was pre-treated with an extract or control antibody (as described for panel A), and then the mixture was incubated with immobilized Gb_3_. The bound DIG-Stx1B was detected with sheep anti-DIG antibodies. The dashed line indicates the absorbance reading without DIG-Stx1B. The concentration of a sample added to each well (abscissa) was expressed as TSP or the IgA concentration determined by sandwich ELISA. For comparison, the IgA concentration is effective for dimer Tg (closed circles) and TEPC 15 (closed squares), and TSP for dimer Tg and wild-type (closed triangles). Data are expressed as means ± SD of triplicate determinations. Error bars underneath the symbols are not visible. The results are representative of three experiments.

The hybrid-IgG/IgA expressed in mammalian cells was shown to bind to Stx1B, and to interfere with the interaction between Stx1B and Gb_3_/CD77-positive Ramos cells [32]. To confirm the function of hybrid-IgG/IgA plantibodies, binding to Stx1B and the ability to inhibit Stx1B binding to Gb_3_ were determined using an ELISA format. Dose-dependent binding of the plantibodies to immobilized Stx1B was observed using anti-α chain as well as anti-κ chain antibodies (Figure 4A). Neither protein extracts of wild-type plants (same protein concentrations as for dimer Tg plants) nor the purified mouse IgA myeloma TEPC 15 exhibited binding activity. The binding of digoxigenin-conjugated Stx1B (DIG-Stx1B) to immobilized Gb_3_ can be detected with anti-DIG antibodies using an ELISA format. When DIG-Stx1B was pre-treated with protein extracts of the dimer Tg plants, the binding of DIG-Stx1B was inhibited in a dose-dependent manner (Figure 4B). Neither protein extracts of wild-type plants nor TEPC 15 inhibited the binding of DIG-Stx1B.

### Prevention of Stx1-induced cell death by the plantibody

**Figure 5:**
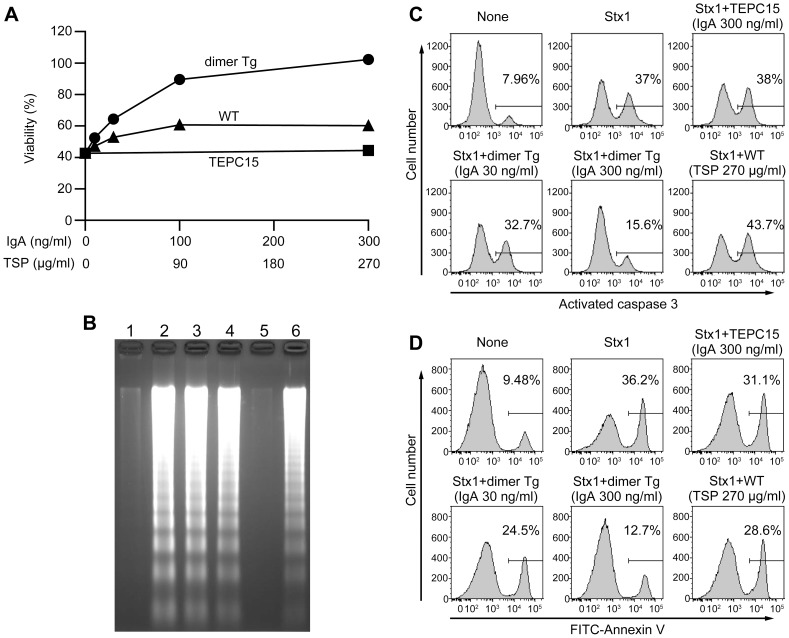
Neutralization of Stx1 holotoxin by the plantibody. Stx1 holotoxin was pre-treated with an extract of dimer Tg plants, an extract of a wild-type plants (WT), or control IgA (TEPC 15) for 1 h, and then the mixture was added to Stx1-sensitive cells. A. Cell viability assay. Vero cells were cultured for 48 h in the presence of 20 pg/ml of Stx1 that had been pre-incubated with a plant extract or control IgA at various concentrations (abscissa). The IgA concentration is effective for Tg and TEPC 15, and TSP for Tg and WT. Cell viability was exhibited as a percentage of the control level (without toxin exposure). Data are expressed as means ± SD of triplicate determinations. Error bars underneath the symbols are not visible. B. DNA fragmentation. Vero cells were cultured for 48 h in the presence of 10 pg/ml of Stx1 that had been pre-incubated with a plant extract or control IgA. DNA ladder formation was observed on agarose gel electrophoresis. Lane 1, untreated; lane 2, Stx1 only; lane 3, Stx1 + TEPC 15 (300 ng/ml IgA); lane 4, Stx1 + Tg (30 ng/ml IgA); lane 5, Stx1 + Tg (300 ng/ml IgA; 270 µg/ml TSP); lane 6, Stx1 + WT (270 µg/ml TSP). C. Caspase 3 activation. Ramos cells were cultured for 5 h in the presence of 10 pg/ml of Stx1 that had been pre-treated as indicated. Activated caspase 3 was probed with FITC-DEVD-FMK (abscissa), and analyzed with a flow cytometer. The number in each graph indicates the percentage of cells with activated caspase 3. D. Annexin V binding. Ramos cells were cultured as described for panel C. The binding of FITC-annexin V (abscissa) revealed cell surface exposure of phosphatidylserine. The number in each graph indicates the percentage of cells labeled with annexin V. The results are representative of three experiments.

Stx1 is known to kill cells expressing Gb_3_/CD77 such as Ramos cells and Vero cells [14], [36]. We examined whether or not the hybrid-IgG/IgA plantibody can prevent Stx1-induced cell death. Upon 48-h exposure to 20 pg/ml of Stx1, approximately 60% of Vero cells were killed, as measured by means of a colorimetric cell viability assay. When Stx1 was pre-incubated with the plantibody, cell viability increased in response to the concentration of the hybrid-IgG/IgA in the dimer Tg plants. Treatment with 300 ng/ml hybrid-IgG/IgA plantibody gave complete inhibition of toxicity (Figure 5A). Neither protein extracts of wild-type plants (same protein concentrations as those in the dimer Tg plants) nor the TEPC 15 myeloma protein (same IgA concentration as those in the dimer Tg plants) caused a sufficient increase in cell viability. As a mechanism underlying the Stx1-induced cytotoxicity, apoptosis induction has been reported [31], [37]. Stx1-treated Vero cells exhibited DNA fragmentation into nucleosomal units (Figure 5B, lane 2). High molecular weight DNA from un-treated cells did not enter the agarose gel and thus was not visible (lane 1). When Stx1 was pre-treated with the plantibody, the DNA fragmentation was inhibited. Treatment with 300 ng/ml of plantibody gave complete inhibition with no DNA fragmentation (lane 5). The DNA fragmentation was not inhibited by extracts of the wild-type plants or the IgA myeloma protein. As one of the intracellular events leading to apoptosis, activation of caspase-3 was examined. Caspase-3 activation was observed in Ramos cells after 5-h incubation with Stx1. Plantibody pre-treatment markedly reduced the percentage of cells with activated caspase-3 (Figure 5C). Caspase-3 activation was not inhibited by extracts of wild-type plants or by the IgA myeloma protein. As another early event of apoptosis, phosphatidyl serine translocation on the plasma membrane was examined in Stx1-treated Ramos cells. Pre-treatment with the plantibody suppressed this event as well (Figure 5D). These results indicate that the plantibody can inhibit Stx1-induced apoptosis of Stx1-sensitive cells.

## Discussion

Treatment of patients with EHEC infection with antibiotics is controversial because there is the risk of the release of large amounts of Stx from dead EHEC leading to life-threatening diseases [4], [6]. Oral administration of Stx-specific IgA is one of the candidate therapies for the treatment of Stx-caused food poisoning. As an important step to produce transgenic plants that can orally deliver Stx-specific IgA on eating, we established transgenic *A. thaliana* expressing Stx1B-specific dimeric hybrid-IgG/IgA, which is responsible for antigen recognition, using a method involving *Agrobacterium*.

In this study, we chose the promoter of *CAB* derived from *A. thaliana* to express hybrid-IgG/IgA in *A. thaliana* (Figure 1A). The CAB protein is one of the highly expressed proteins in plant leaves, and the *CAB* promoter expresses two proteins bi-directionally [38]. Thus, we expected concurrent expression of the hybrid-IgG/IgA heavy and light chains with high expression efficiency. On analysis of the expression levels of hybrid-IgG/IgA by means of sandwich ELISA, the amount of hybrid-IgG/IgA was determined to be 11 μg/g leaf tissue (0.11% of the total soluble protein) (Figure 3B). This expression level is comparable to those in reports of expression of plantibodies using commonly used promoters such as the cauliflower mosaic virus *35S* promoter [39]. Regarding this expression level, the cost of antibody production by plants was calculated to be of the order of $7.9 per mg hybrid-IgG/IgA. In contrast, the production cost for hybrid-IgG/IgA using COS-1 cell cultures was calculated to be $630 per mg in our laboratory. This calculation is consistent with previous studies showing that a plant expression system is more cost-effective than a mammalian cell system [23], [25]. It has been reported that the optimization of codon usage for plants or the addition of the KDEL tetra-peptide signal for ER retention can increase protein expression and accumulation [40]–[42]. Such modifications of the *hybrid-IgG/IgA* genes may increase the expression and accumulation of plantibodies, and give rise to more cost-effective production. Our data suggest that the *CAB* promoter is useful for expressing IgA genes in a single step.

SIgA consists of two monomeric IgAs that are linked by a J chain and an attached SC. The J chain is required for the dimer formation of IgA, which is a prerequisite for the formation of SIgA [43]. Immunoblot analysis indicated that dimeric hybrid-IgG/IgA with the J chain could be assembled in *A. thaliana*, though several other forms were also present in leaf extracts (Figure 2A). In addition, an un-known band was observed that did not match the molecular mass of any form of hybrid-IgG/IgA under reducing conditions (Figure 2B). One possibility is proteolytic degradation of hybrid-IgG/IgA in *A. thaliana*. In previous studies involving tobacco, plantibodies were shown to be digested by proteases in vacuoles and apoplasts [44], [45]. Another possibility is the production of incomplete antibodies due to the differences in codon usage between plants and animals. During heterologous protein expression, differences in synonymous codon usage may lead to translational frame-shifts, early translation termination or misfolding [40], [46], [47]. The codon frequency of the mouse hybrid-IgG/IgA genes is different from that of *A. thaliana*. For example, CTG is used as 58.5% of the leucine codons, and 26 out of 477 total codons (5.5%) are CTG in hybrid-IgG/IgA heavy chain genes. In contrast, CTG is used as 10.5% of the leucine codons, and 9.8 per thousand triplets form CTG in the *A. thaliana* genome. Further studies may be needed to clarify these points for more efficient production and stable assembly of plantibodies.

The hybrid-IgG/IgA plantibody exhibited dose-dependent binding to immobilized Stx1B (Figure 4A). Pre-treatment with the plantibody effectively inhibited the binding of DIG-Stx1B to immobilized Gb_3_ (Figure 4B). The dose–response curve of the inhibition of DIG-Stx1B binding was shown to exhibit an inverse relationship to the curve of the plantibody binding. The results indicated that the plantibody was capable of interfering with the carbohydrate binding of Stx1B.

We then examined the ability of the hybrid-IgG/IgA plantibody to neutralize the cytotoxic effect of Stx1 on Vero cells and Ramos cells. Stx1 is known to induce apoptosis in several Gb_3_-positive cell lines including Burkitt’s lymphoma cell lines and Vero cells [14], [37]. We confirmed that Stx1 inhibited cell viability and that it induced apoptosis of Gb_3_-positive cells (Figure 5). Consistent with the finding that the plantibody effectively inhibited the binding of DIG-Stx1B to Gb_3_, the plantibody efficiently inhibited death as well as apoptosis of Gb_3_-positive cells. These results indicate that the plantibody can neutralize Stx1 *in vitro*.

A crude extract of the dimer Tg plants gave several bands recognized by anti-α antibodies other than the band representing dimeric IgA (Figure 2A). It is not conclusive that the dimeric IgA is solely responsible for the toxin neutralization in the plant extract at present. However, comparison with transgenic plants expressing H and L chains alone revealed that the content of monomeric IgA appears to be negligible in the plant extracts expressing H, L and J chains together. Further comparison with anti-κ and anti-J immunoblots revealed molecules with single H and L chains as candidates that could retain the biological function. Otherwise, such molecules do not retain binding activity despite that they have both heavy and light chains. The latter situation may lead to overestimation of the content of biologically active antibodies in plant extracts. To solve this question, purification of the dimeric IgA is in progress.

In conclusion, we established transgenic *A. thaliana* that expresses dimeric hybrid-IgG/IgA specific for Stx1B using the CAB promoter by single step transformation. Dimeric hybrid-IgG/IgA was shown to be assembled in *A. thaliana*. The hybrid-IgG/IgA plantibody retained binding activity as to Stx1B, inhibited the binding of Stx1B to Gb_3_, and neutralized Stx1-induced cytotoxicity toward Vero cells and Ramos cells. *A. thaliana* expressing SIgA specific for Stx1B can be obtained by crossing the present *A. thaliana* and a transgenic strain expressing SC. The resulting *A. thaliana* could be used to study the immune exclusion process on oral administration of transgenic plants. Such study will provide useful information on the role of antigen-specific SIgA *in vivo.* Due to the low production cost, an *in vivo* animal study involving plantibodies would be a more cost-effective strategy than one involving recombinant antibodies produced by animal cell cultures. It is also important that plantibodies with any specificity can be made by changing variable regions. Such plantibodies may be useful for antibody therapy against a variety of agents that enter through mucosal surfaces.

## Supporting Information

Table S1 Primers used for PCR reactions.
(PDF)

Method S1 **Expression of monomeric hybrid-IgG/IgA in** ***A. thaliana*** **.**
(PDF)
