# Supplementary material for: Production of Hybrid-IgG/IgA Plantibodies with Neutralizing Activity against Shiga Toxin 1
Source: PLoS One. 2013 Nov 28;8(11):e80712. doi: 10.1371/journal.pone.0080712 (PMC3842918; doi:10.1371/journal.pone.0080712)
Supplement: Table S1 — Primers used for PCR reactions. (PDF) [file pone.0080712.s001.pdf]

Table S1. Primers used for PCR reactions.

Gene	Name of primers	DNA sequence
CAB terminator	TCAB1 fd *Not*	5′-CAGCGGCCGCTTCGTGAGTGTGAGAG-3′
CAB terminator	TCAB1 rv *Nsi*	5′-TGTATGCATTGTGGAAGCTTGCTCACATCA-3′
CAB terminator	TCAB2 fd *Sac*	5′-GCCGAGCTCTATCTGTTTGTAATTTACTTTT-3′
CAB terminator	TCAB2 rv *Nsi*	5′-CATGAATTCACCCATGCATTGGA-3′
CAB promoter	PCAB2F2-*Sac*II	5′-TTGTTGAGGCCCGCGGTGAAACTTTTTTGTGTT-3′
CAB promoter	PCAB1R2-*Sac*II	5′-TTGAGGCCCGCGGTGAGGTTGAGTAGTGC-3′
J chain	JCF-*Xba*	5′-ACAGTCTAGACAAGATGAAG-3′
J chain	JCR-*Xho*	5′-GTGCTGGATATCTCGAGAAT-3′
J chain	JCR	5′-CTAGTCAAGGTAGCAAGAAT-3′
IgG H chain	IgG Heavy *Not*F	5′-CACTGCGGCCGCTGACTCTAACCATGGGATGGAGC-3′
IgA H chain	IgA-H/*Not*R	5′-GGGCGGCCGCTCAGTAGCAGATGCCATCTCCCTC-3′
IgG L chain	IgGk *Not*F	5′-TGTGCGGCCGCAGCAGAAACATGAAG-3′
IgG L chain	IgGk *Not*R	5′-CGAGCGGCCGCTTCTAACACTCATTCC-3′
ACTIN2	actin2-F	5′-CTTACAATTTCCCGCTCTGC-3′
ACTIN2	actin2-R	5′-GTTGGGATGAACCAGAAGGA-3′
