# Supplementary material for: Production of Hybrid-IgG/IgA Plantibodies with Neutralizing Activity against Shiga Toxin 1
Source: PLoS One. 2013 Nov 28;8(11):e80712. doi: 10.1371/journal.pone.0080712 (PMC3842918; doi:10.1371/journal.pone.0080712)
Supplement: Method S1 — Expression of monomeric hybrid-IgG/IgA in A. thaliana . (PDF) [file pone.0080712.s002.pdf]

Method S1. Expression of monomeric hybrid-IgG/IgA in *A. thaliana*.

Vector construction

To produce monomeric hybrid-IgG/IgA in *Arabidopsis thaliana*, two binary vectors were constructed. The cDNA fragments encoding hybrid-IgG/IgA Hc and Lc were amplified by PCR using the primer set of p-hybridHc-F2/p-hybridHc-R2 and p-IgGk-F1/p-IgGk-R1, respectively. Amplified DNA fragments were digested with *Sma*I/*Xho*I or *Sac*I/*Xba*I, and then ligated into the respective sites of pBCH1 or pSMAB704 [1], resulting in the construction of pBCH1/Hc and pSMAB704/Lc binary vectors.

Production of transgenic *A. thaliana* expressing monomeric hybrid-IgG/IgA

pBCH1/Hc or pSMAB704/Lc was introduced into *Agrobacterium tumefaciens* GV3101 by electroporation using a Gene Pulser II (Bio-Rad). Wild-type *A. thaliana* (ecotype Col-0) plants were grown in a temperature-controlled room with 24 h light at 20 ºC for 6 wk. *A. thaliana* plants were transformed with the *Agrobacterium* harboring pSMAB704/Lc via the floral dip method. The primary transformants were selected on MS medium supplemented with 20 μg/ml glufosinate ammonium. Four weeks after seeding, glufosinate-resistant *A. thaliana* plants were transferred to soil in pots and then grown under the same conditions as for the wild-type plants for 2wk. The glufosinate-resistant *A. thaliana* plants were then transformed with the *Agrobacterium* harboring pBCH1/Hc. The double-transformants were selected on MS medium supplemented with 20 μg/ml hygromycin B and 20 μg/ml glufosinate ammonium. After selection, *A. thaliana* plants expressing monomeric hybrid-IgG/IgA were transferred to

soil in pots and then grown. The recombinant plants were grown within the physical containment level 1-plant (P1P) facility of the University of Shizuoka.

Supplementary reference

1. Nishihara M, Nakatsuka T, Hosokawa K, Yokoi T, Abe Y, et al. (2006) Dominant inheritance of white-flowered and herbicide-resistant traits in transgenic gentian plants. Plant Biotechnology 23: 25–31.

Primers used for PCR reactions

Gene	Name of primers	DNA sequences
H chain	p-hybridHc-F2	5′-TGGCCCCGGGTGACTCTAACCATGGGA-3′
H chain	p-hybridHc-R2	5′-AGACTCTCGAGGCCGCTCAGTAGCA-3′
L chain	p-IgGk-F1	5′-CGCTCTAGAAACATGAAG-3′
L chain	p-IgGk-R1	5′-AGCGAGCTCTTCTAACAC-3′
